# Should chlorhexidine mouthwash be a concern for blood pressure in dental patients?

**DOI:** 10.1038/s41432-026-01233-z

**Published:** 2026-07-08

**Authors:** Mahdi Mutahar, Akram Alshirah, Essam Ahmed Al-Moraissi

**Affiliations:** 1 School of Dentistry, Health and Care Professions, University of Portsmouth, Portsmouth, UK; 2 Faculty of Dentistry, The Hashemite University, Zarqa, Jordan; 3 Department of Oral and Maxillofacial Surgery, Faculty of Dentistry, Thamar University, Thamar, Yemen

**Keywords:** Periodontics, Diseases

## Abstract

**A Commentary on:**

**Toonen LSJ, et al**.

The Effect of Chlorhexidine Mouthwash on Blood Pressure: A Systematic Review and Meta-Analysis. Int J Dent Hyg. 2026. https://doi.org/10.1111/idh.70035.

**Objective:**

To evaluate whether current clinical evidence supports an association between chlorhexidine mouthwash (CHX-MW) use and changes in blood pressure in human populations.

**Data sources:**

Evidence was derived from a systematic review and meta-analysis of studies identified through PubMed/MEDLINE and Cochrane CENTRAL up to December 2024.

**Study selection:**

Randomised, controlled, and observational studies evaluating blood pressure following chlorhexidine mouthwash use were included.

**Methods:**

Evidence was synthesised through narrative review and meta-analysis, with methodological quality and risk of bias assessed using established appraisal frameworks.

**Results:**

Five studies met the inclusion criteria. Although two studies reported small increases in blood pressure, the pooled analysis found no statistically or clinically significant effect of chlorhexidine mouthwash on systolic or diastolic blood pressure.

**Conclusions:**

Current evidence does not demonstrate a clinically meaningful effect of chlorhexidine mouthwash on blood pressure, but certainty remains low due to methodological limitations and indirectness of study populations.

## GRADE Rating:



## Commentary

(CHX-MW) is widely used in dentistry for plaque control, gingivitis, postoperative care, and periodontal therapy, particularly when mechanical cleaning is limited^1,2^. Its strong antimicrobial activity and substantivity have established it as a standard adjunct in practice^1,2^. However, emerging microbiome evidence suggests antiseptic mouthwashes may have systemic effects through disruption of the nitrate–nitrite–nitric oxide pathway, where oral bacteria convert dietary nitrate into nitrite, contributing to nitric oxide production and blood pressure regulation^3,4^. The authors of the systematic review and meta-analysis^5^ are commended for adherence to PRISMA 2020 guidelines, protocolised methodology, and transparent reporting; however, key methodological limitations may weaken the certainty of their conclusions. Their work has directly addressed whether rinsing with CHX-MW affects systolic blood pressure (SBP) and diastolic blood pressure (DBP) in humans^5^. The authors included randomised controlled trials, controlled clinical trials and observational studies, and concluded that any increase in blood pressure among normotensive users is likely to be insignificant or very small. They identified five eligible studies^5^: Kapil et al., Sundqvist et al., Tribble et al., Bescos et al. and Bescos et al.^3,6–9^. The review was prospectively registered in PROSPERO and reported according to PRISMA and Cochrane guidance^5^. The included evidence was mixed: two studies reported increases in SBP and DBP following CHX-MW use^3,8^, whereas three did not show significant effects^6,7,9^. The pooled meta-analysis showed no statistically significant difference for SBP (1.56 mmHg, 95% CI −0.67 to 3.80) or DBP (−0.10 mmHg, 95% CI −1.99 to 1.78)^5^. However, substantial clinical heterogeneity existed across study design, CHX concentration, duration of exposure, dietary nitrate control and BP measurement methods^3,5–9^. Biological plausibility remains important. CHX-MW can reduce nitrate-reducing oral bacteria, potentially disrupting nitric oxide production, which is involved in vascular regulation^3,4^. The key question is therefore not whether the mechanism exists, but whether its magnitude is clinically meaningful during routine dental use. The review is clinically relevant because CHX is widely prescribed and often used without supervision. If antimicrobial mouthwash alters nitrate-reducing bacterial activity, even small BP effects may be relevant in cardiovascular risk populations. The authors used a structured methodology, including a clear PICOS question, protocol registration, duplicate screening and systematic synthesis^5^. However, only five studies were included, limiting precision and subgroup analysis.

Participants were predominantly healthy, normotensive adults, limiting external validity to hypertensive, elderly or medically complex patients. Intervention durations were short (3–7 days), which does not reflect prolonged or repeated real-world use^3,6–9^. The pooled BP change was small and close to measurement error thresholds, supporting the conclusion that short-term effects are likely clinically negligible in healthy individuals^5^. However, caution remains warranted. Significant findings in individual studies should not be dismissed as they align with a biologically plausible mechanism^3,8^. The most balanced interpretation is that CHX-MW appears safe for short-term indicated use in normotensive adults, but uncertainty remains regarding repeated exposure and high-risk populations. External validity is also limited because CHX is primarily used in patients with gingivitis, periodontitis or post-surgical oral conditions rather than healthy volunteers. These clinical populations differ significantly in inflammatory burden, microbiome composition and systemic risk profile. Therefore, evidence from healthy participants may not fully represent real clinical scenarios^1,2,5^. RoB 2 assessment of randomised trials also indicates some concerns due to small sample sizes, potential blinding limitations and variability in BP measurement^10^. ROBINS-I considerations for non-randomised studies highlight confounding from diet, oral health status, stress, medication use and time-related effects^3,8^. AMSTAR 2 appraisal suggests moderate confidence in the review overall, although limited database searching and small study numbers reduce certainty^11^.

In conclusion, CHX-MW appears unlikely to cause clinically meaningful BP increases in short-term use among healthy adults, a finding supported by unpublished crossover RCT in healthy individuals and people with dental erosion (Mutahar et al., manuscript in preparation), which also found no significant effects on systolic or diastolic blood pressure. However, the evidence is limited by small sample sizes, short follow up periods, and heterogeneity between studies, and does not adequately address long-term or high-risk populations. Chlorhexidine should therefore be used when clearly indicated and for limited durations, until further research in clinically relevant populations provides more definitive evidence.

Practice Points

- Current evidence but certainty is very low; does not show a clinically meaningful effect of short-term chlorhexidine mouthwash on blood pressure, dentists should therefore avoid assuming cardiovascular safety or harm based on this evidence alone.
- In routine dental practice, chlorhexidine should be prescribed only when clearly indicated (e.g., acute gingival inflammation, post-surgical care) and for the shortest effective duration, particularly in patients with hypertension or cardiovascular disease.
- Dentists should remain cautious when prescribing repeated or prolonged courses in medically complex patients, as current evidence is based mainly on healthy individuals and does not adequately represent typical high-risk dental populations.
- Further well-designed, adequately powered, placebo-controlled RCTs with standardised protocols and clinically relevant patient populations are required before firm conclusions on cardiovascular safety can be made.
